# Synthetizing Published Evidence on Survival by Reconstruction of Patient-Level Data and Generation of a Multi-Trial Kaplan-Meier Curve

**DOI:** 10.7759/cureus.19422

**Published:** 2021-11-09

**Authors:** Andrea Messori

**Affiliations:** 1 Health Technology Assessment (HTA) Unit, Regione Toscana, Firenze, ITA

**Keywords:** immune checkpoint inhibitors, meta-analysis, reconstruction of patient-level data, kaplan-meier survival curves, individual-patient data

## Abstract

Introduction

In conducting a survival meta-analysis, the typical methodological approach analyses the hazard ratios (HRs) of individual trials and then combines them into a pooled meta-analytical estimate. The length of follow-up of individual trials is not generally accounted for. Recent techniques aimed at individual patient-data reconstruction from Kaplan-Meier graphs represent an important methodological innovation. These techniques permit the combination of the survival curves published in a single clinical trial but are also applicable to more than one trial. In the case of multiple trials, a meta-analysis can be conducted without using any statistical model of meta-analysis.

Methods

As an example of this new approach, we applied a technique of individual patient data reconstruction to the Kaplan-Meier graphs of overall survival reported in two phase-III trials, which were conducted on patients with locally advanced/advanced non-small cell lung cancer selected according to their PD-L1 expression status, not previously treated for their metastatic disease. Only subjects with PD-L1 ≥50% were considered for our analysis. The experimental arms received pembrolizumab monotherapy while the control arms were given platinum-based chemotherapy. The survival graphs were obtained for both trials. For each Kaplan-Meier curve, the graph was firstly digitalized. Then, the Shiny package was used to reconstruct patient-level data. Finally, the pooled survival curves were generated from the reconstructed patient-level data along with the relevant Cox statistics; for this purpose, we used three packages (“coxph”, “survfit”, and “ggsurvplot”) under the R-platform.

Results

In our pooled analysis based on this procedure, we compared 453 patients given pembrolizumab vs. 451 controls given chemotherapy. The HR estimated from reconstructed patient-level data was 0.670 (95% confidence interval [CI], 0.566 to 0.793).

Conclusion

The analysis described herein demonstrates the easy applicability of the Shiny technique. This technique was successful in generating a pooled survival graph for the experimental treatment groups vs. controls and efficiently estimated the pooled HR in which the results of the two trials were combined.

## Introduction

The methodology of survival meta-analysis is complex [1]. The approach most commonly used analyzes the hazard ratios (HRs) of individual trials and then combines them into a pooled meta-analytical estimate. One drawback of this approach is that it does not account for the length of follow-up of individual trials. Another is that it is not applicable to one-arm trials. Finally, HR has some intrinsic disadvantages compared with other more sophisticated but complex parameters (such as the restricted mean survival time [2]).

In recent times, techniques that reconstruct individual patient data from the graphs of Kaplan-Meier curves have considerably improved in terms of performance and easy applicability [3]. One advantage is that the availability of these techniques permits the combination of multiple survival curves published in different trials without using any meta-analytical statistics.

## Materials and methods

**Figure 1:**
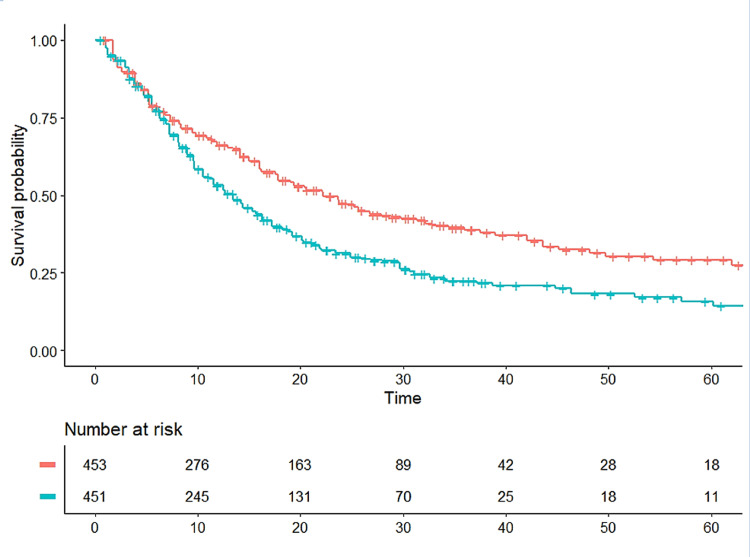
Kaplan-Meier curves from reconstructed patient-level data Pooled Kaplan-Meier survival curves obtained by reconstruction of individual patient data from two trials (KEYNOTE-024 [4] and KEYNOTE-042 [5]). See text for details. Treatment groups, in red; time expressed in months.

As an example of this approach, we applied the Shiny technique of individual patient data reconstruction [3] to the Kaplan-Meier graphs of overall survival reported in the KEYNOTE-024 [4] and KEYNOTE-042 [5] trials. Both trials were phase-III and were conducted in patients with locally advanced/advanced non-small cell lung cancer selected according to their PD-L1 expression status, not previously treated for their metastatic disease, and receiving first-line PD-(L)1 monotherapy. Only subjects with PD-L1 ≥50% were considered for our analysis. The experimental arms received pembrolizumab monotherapy while the control arms were given platinum-based chemotherapy. The survival graphs were obtained from Figure 1 for KEYNOTE-024 (154 *vs.* 151 patients; follow-up of 66 months; 226 deaths) and for KEYNOTE-042 (299 vs.300 patients; follow-up of 40 months; 356 deaths). For each Kaplan-Meier curve, the graph was digitalized and converted into x-y data pairs using Webplotdigitizer (Version 4.5, URL: https://apps.automeris.io/wpd/); then, the Shiny package (Version: 1.2.2.0; subprogram “Reconstruct Individual Patient Data”; URL: https://www.trialdesign.org/one-page-shell.html#IPDfromKM, see Reference [3]) was used to reconstruct patient-level data on the basis of the x-y data pairs, the total number of enrolled patients, and the total number of events. Finally, the pooled survival curves were generated from the reconstructed patient-level data along with the relevant Cox statistics; for this purpose, we used three packages (“coxph”, “survfit”, and “ggsurvplot”) under the R-platform.

## Results

In our pooled analysis based on this procedure, we compared 453 patients given pembrolizumab vs.451 controls given chemotherapy. Figure 1 shows the two pooled Kaplan-Meier curves. The HR estimated from reconstructed patient-level data was 0.670 (95% confidence interval [CI], 0.566 to 0.793). This HR resulting from a combined analysis of the two trials cannot be compared with a “true” value because the authors of the two trials have not carried out any pooled analysis nor have they published any estimate of this HR.

On the other hand, as regards the KEYNOTE-024 trial, the HR that we estimated from our reconstructed data (HR=0.62350; 95%CI, 0.472243 to 0.817535) was virtually identical to that reported in the original trial (HR=0.62; 95%CI, 0.48 to 0.81). We found the same result for the KEYNOTE 042 trial too (HR from reconstructed data: 0.703482; 95%CI: 0.568048 to 0.871206; HR from original data = 0.69; 95%CI, 0.56 to 0.85). It is not surprising that the statistical results based on reconstructed data were so close to the original results because the Shiny procedure is known to have an excellent performance.

## Discussion

When two or more randomized trials are available on a therapeutic issue and the clinical end-point is expressed as time-to-event, synthetizing the clinical evidence is a complex issue, and there is presently no consensus on which methodological approach should be preferred [1,6]. Pooling the values of HR is certainly the method most commonly used, but its important limitations have been widely recognized for many years (e.g. the inability to account for the length of follow-up, the inability to model variations of risk over time, the dimensionless nature of HR as opposed to the greater informative value of absolute parameters such as medians, etc.) [2]. The development of the restricted mean survival time (RMST) has represented an advancement in this field, but the use of this parameter, unfortunately, remains low.

In this context, the marked improvement in the performance of techniques that reconstruct individual-patient data [3] represents an important innovation, the role of which still needs to be fully evaluated. On the one hand, reconstructing individual-patient data is a mandatory prerequisite to determine the RMST, and this explains the increased use of these reconstruction techniques when a single trial has been analyzed through the RMST [2]. On the other hand, another potential use of these techniques is increasingly being recognized in the analysis of multiple trials; in such cases, these techniques offer a new methodological alternative to standard meta-analytic methods [1] and to the more recent approaches where meta-analysis is based on the use of RMSTs [7-8].

The various parameters mentioned above (especially HR, RMST, and median) have been investigated for many years to identify their respective advantages and disadvantages, and the literature on this issue is wide [2]. In contrast, the literature on the use of reconstructed survival curves is still in its early stages [3], and this applies particularly when multiple trials are analyzed and pooled together.

The experience described herein has been aimed at offering a limited but useful contribution to the development of meta-analysis-like methods based on reconstructed survival curves.

## Conclusions

The example described herein demonstrates the feasibility of reconstructing patient-level data from survival graphs to generate survival statistics from these reconstructed data and synthetize the clinical evidence. This paper has been specifically focused on the case where patient-level data reconstruction is applied to multiple trials so that this methodological innovation represents an alternative to standard survival meta-analysis. To evaluate the advantages and disadvantages of this approach, further analyses will be needed to compare reconstructed statistical results with those published originally.
